# Photobiomodulation Therapy vs. Corticosteroid for the Management of Erosive/Ulcerative and Painful Oral Lichen Planus. Assessment of Success Rate during One-Year Follow-Up: A Retrospective Study

**DOI:** 10.3390/healthcare9091137

**Published:** 2021-08-31

**Authors:** Samir Nammour, Marwan El Mobadder, Aldo Jr. Brugnera, Melanie Namour, Saad Houeis, Daniel Heysselaer, Alain Vanheusden, Amaury Namour

**Affiliations:** 1 Department of Dental Science, Faculty of Medicine, University of Liège, 4000 Liège, Belgium; marwan.mobader@gmail.com (M.E.M.); melanienamour@gmail.com (M.N.); saad.houeis@gmail.com (S.H.); daniel.heysselaer@gmail.com (D.H.); alain.vanheusden@chu.ulg.ac.be (A.V.); amaurynamour@gmail.com (A.N.); 2 Nacional Institute of Science, IFSC-USP, São Carlos 04026-001, Brazil; aldo.brugnera@gmail.com

**Keywords:** lichen planus, chronic inflammatory oral disease, laser therapy, oral laser applications, photobiomodulation, low-level laser therapy

## Abstract

Photobiomodulation (PBM) therapy is a promising approach for the management of inflammatory conditions and autoimmune lesions, such as oral lichen planus (OLP). The aim of this retrospective study was to assess the effectiveness of PBM in the management of painful and erosive/ulcerative OLP and to compare it with the standard of care that is the topical application of corticosteroids. 96 patients were included with erosive and painful OLP. 48 patients received PBM therapy and 48 received corticosteroids. Data was collected retrospectively on pain using the visual analogue scale; clinical aspects of lesions were assessed with the REU score, and the recurrence rate was noted. One session of PBM therapy with a helium-neon red light (635 nm) was carried out every 48 h for 6 weeks. Treatments were mainly made in contact mode, using a fiber with a diameter of 600 µm (0.6 mm). The output power of the laser beam was calibrated by a power meter. A delivered power of 0.1 W was used for 40 s in a continuous wave (CW), corresponding to a delivered energy of 4 J. The delivered energy density related to the fiber diameter was 1415 J/cm^2^. Each treated point was considered as 1 cm^2^ of diameter. PBM therapy within these parameters was carried out on each point until the totality of the lesion was covered, including the non-erosive OLP area. Furthermore, healthy mucosa within 5 mm of the lesion was also irradiated with the same conditions. This PBM treatment was performed during 6 consecutive weeks. The topical corticosteroid treatment consisted of cortisone application to cover the OLP 3 times/day for 6 weeks. Follow-up was made at 6 weeks and at 3, 6 and 12 months. After 6 weeks, both groups showed complete absence of pain, and a complete disappearance of ulcerative/erosive areas. No significant difference was found for both groups concerning the recurrence rate of erosive OLP during the follow-up period; values were 0% at 6 weeks for both groups and 79% and 87.5% for the corticosteroid and PBM group, respectively, at 12 months of follow-up. PBM is effective for managing OLP and is significantly similar to topical corticosteroids without any need for the use of medication and with no reported side effects.

## 1. Introduction

Photobiomodulation (PBM) therapy, previously known as low-level laser therapy, is the therapeutic use of light in order to modulate biological activity [1]. The North American Association of Laser Therapy (NAALT) and the World Association of Laser Therapy (WALT) reached a consensus in 2014 on adopting the term photobiomodulation instead of low-level laser therapy [1]. It is now well-established that PBM therapy can be effective in numerous indications, such as in the management of oral inflammation due to high-dose chemotherapy and/or head and neck radiotherapy in cancer patients, as an assistance in tempero-mandibular joint disorders, and other indications [1,2]. The exact mechanism of action of PBM is not fully understood; however, it is now well-established that PBM acts primarily by increasing adenosine triphosphate (ATP) production and causing a short transient burst of reactive oxygen species, which have a beneficial impact on the inflammatory process [1,2,3]. The most acceptable theory is that in certain conditions, red and infrared light can stimulate cytochrome c oxidase, which leads to an increase in ATP production. In addition, recent studies have suggested that PBM may activate transcription factors and signaling pathways and may have a protective mechanism [3,4].

Lichen planus (LP) is a common chronic autoimmune lesion that can affect skin and mucous membranes, including the oral mucosa. Lichen planus presents with characteristic relapses and remissions that can be a source of morbidity and can present a rare but possible malignant transformation [5,6].

The management of symptomatic oral lichen planus (OLP) differs significantly. Choices vary based on the elimination of the precipitating or provoking factors—local or systemic, psychosocial interventions or long-term pharmacological therapies [5,6]. Local application of corticosteroids is still considered the treatment of choice for the management of OLP. However, promising approaches are being studied with promising positive results. For example, a study by Bennardo, F. et al. showed that platelet-rich fibrin can be effective in reducing the extension and symptomatology of OLP lesions with similar results to those obtained with the topical application of steroids [7].

The prevalence of OLP is estimated to be 0.5 to 2% in the adult population, with a reported female/male sex ration of 2/1 and an age of onset between 30 and 60 years [5,6,7,8]. Cutaneous and genital lichen planus are related to almost 15 and 20% of oral cases, respectively, while it is estimated that OLP occurs in 70 to 77% of patients with cutaneous lichen planus [8]. The exact etiology of OLP is still poorly identified, but several predisposing factors were described in the literature, such as genetic background, hepatitis C virus, hypertension, diabetes mellitus, trauma and psychological factors. Clinically, OLP can be classified into six different variants: reticular (fine white striae cross each other in the lesion), atrophic (areas of erythematous lesion surrounded by reticular components), papular type, bullous type, plaque type, and the erosive or ulcerative type [8].

According to the World Health Organization (WHO), the diagnosis of OLP remains histological. This histological aspect is characterized by the presence of a thickened ortho- or para-keratinized layer in sites that are normally keratinized. If the sites are normally non-keratinized, this layer may be thin, with the presence of civatte bodies in the basal layer, the epithelium, and the superficial part of connective tissue, the presence of a well-defined band-like zone of cellular infiltration that is confined to the superficial part of the connective tissue and consists mainly of lymphocytes, and signs of liquefaction degeneration in the basal cell layer [9].

Several studies have shown that PBM can be an effective treatment in autoimmune and chronic inflammatory conditions and stressed cells [10] by attenuating and/or reducing the inflammatory process and promoting wound healing and tissue regeneration [3,4,5,6,7,8,9,10,11].

Concerning the use of PBM for the treatment of OLP, studies are still limited with poor description of the exact PBM procedures and parameters and with a short-term follow-up. However, a systematic review and meta-analysis by Wang B. et al. [12] showed that the treatment of oral lichen planus with PBM could be a reliable alternative to topical corticosteroids with no or less severe complications in a short-term period. However, it was concluded that further investigations are still necessary [12].

Furthermore, any new treatment able to avoid the use of medications for systematic oral pathologies is encouraged to avoid the side effects of prolonged intake of medications such as corticosteroids.

Therefore, the aim of this multi-center long-term retrospective study is to assess the effectiveness of a suggested PBM protocol vs. conventional therapy with corticoids for the management of erosive and painful OLP. The null hypothesis was that PBM therapy will not have a significant impact on OLP.

## 2. Materials and Methods

### 2.1. Study Design

Our multicenter retrospective study was conducted using data collected in the period from 2012 to 2020. Data collection was carried out for all patients with erosive and painful OLP diagnosed clinically and confirmed by a histopathological examination who were treated with one of the following methods: conventional corticoid treatment or photobiomodulation therapy. Moreover, according to the ethical committee recommendations of our university hospitals, the decision for PBM treatment and/or conventional treatment was made after informing all patients about the steps of the treatment, as well as the possibility of a failure and/or recurrence. We only collected clinical cases in which PBM and conventional therapies were performed on patients who had received and signed a written informed consent and, subsequently, an analysis of data collected was made. Our study cannot be considered to be a new clinical study and therefore did not require legally a prior approval from the ethical committee of the University of Liege.

### 2.2. Participants

A total of 96 patients participated in this retrospective study; the mean age of the patients was 48 (minimum 42 and maximum 68), with 66.67% females (*n* = 64) and 33.33% males (*n* = 32) (Table 1). We collected the data of two groups. Forty-eight patients received the conventional treatment protocol consisting of the use of corticosteroids (corticosteroids group; *n* = 48). The other forty-eight patients received PBM therapy (photobiomodulation group; *n* = 48). The data was retrospectively entered into the database, including patient demographics (age, gender, dimension of the lesion). The follow-up periods for the effectiveness of the treatment in terms of recurrence rates were carried out at 6 weeks, 1 month, 6 months, and 12 months after treatment.

### 2.3. Inclusion and Exclusion Criteria

**Figure 1:**
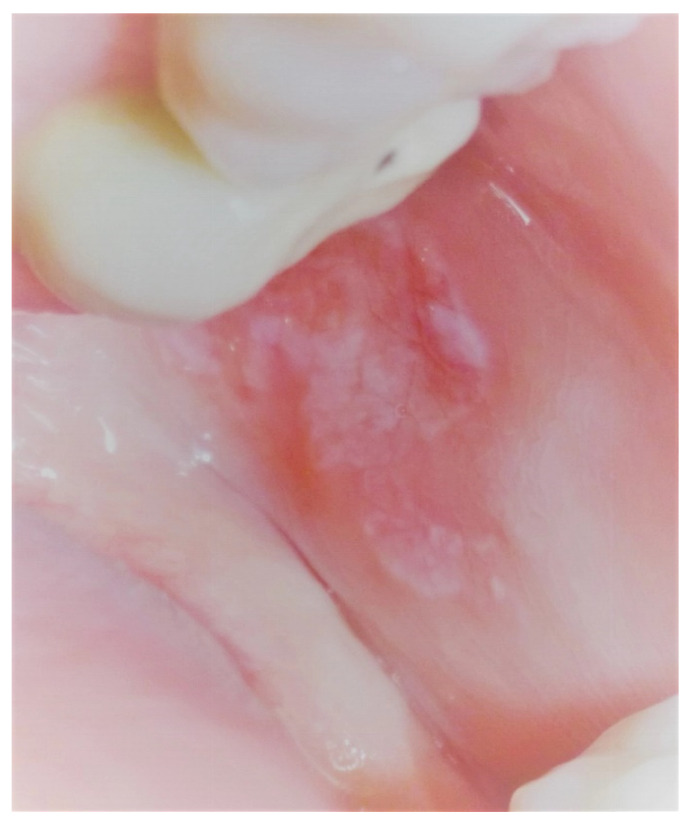
Clinical aspect of the erosive OLP on cheek.

Patients that were diagnosed with erosive and painful OLP (Figure 1), confirmed by biopsy according to the classification of the WHO, who were seeking treatment and signed the written informed consent were included in the study.

#### Exclusion criteria were as follows:

- Pregnant or breastfeeding woman;
- Patients who were having any other treatment for OLP;
- Patients who had used anti-inflammatory drugs (topic or systemic) in the last 30 days;
- Patients who reported drug-related development of oral lichenoid lesions, including imatinib, methyldopa, IFN-alpha and/or infliximab;
- Patients with an uncontrolled systemic disease;
- OLP with epithelial dysplasia or malignant transformation in the histopathological evaluation.

### 2.4. Treatment of Oral Lichen Planus by Topical Medication

Forty-eight participants (*n* = 48) in this retrospective study received only a conventional treatment of OLP that consisted of local application of cortisone. Instruction on how to apply topical cortisone (clobetasol propionate gel 0.05%) was made. The instructions involved cortisone application to cover the OLP lesions completely, three times/day for 6 weeks.

### 2.5. Photobiomodulation Therapy (PBM Group)

**Figure 2:**
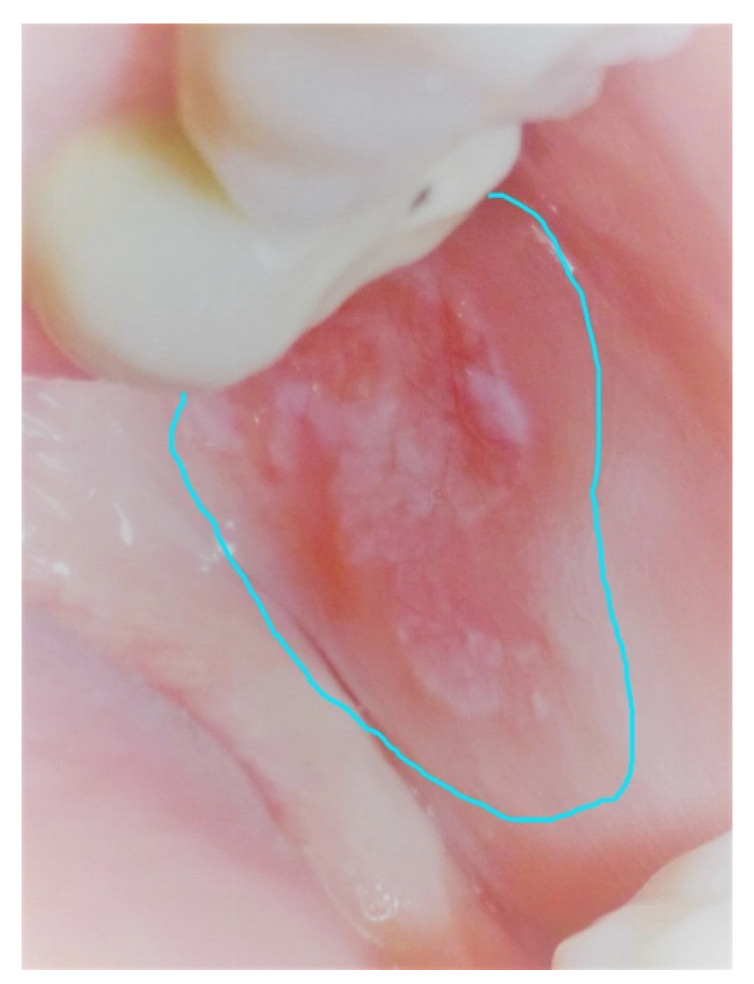
The limit of the area treated by PBM including the healthy area of the mucosa is drawn in a blue line.

For the PBM group (*n* = 48), after giving the proper oral hygiene instructions, PBM therapy was made each 48 h for 6 weeks. The treatment consisted of one session of PBM therapy with a laser helium-neon (He-Ne) red light. The He-Ne laser emitted at 635 nm (Laser Biophoton, Biophoton Inc., Saint Alban, France). Treatments were made mainly in contact mode using a fiber with a diameter of 600 µm (0.6 mm). The output power of the laser beam was calibrated by a power meter (model Tuner, Genstar Technologies Company, Inc, Chino, CA, USA). The delivered power of 0.1 W was used during 40 s in a continuous wave (CW), corresponding to a delivered energy of 4 J. The delivered energy density related to the fiber diameter was 1415 J/cm^2^. Each treated point was considered to be 1 cm^2^ of diameter. PBM therapy within these parameters was made on each point until the totality of the lesion was covered, including the non-erosive OLP area. Furthermore, healthy mucosa within 5 mm surrounding the lesion was also irradiated with the same conditions (Figure 2). This PBM treatment was performed during 6 consecutive weeks.

### 2.6. Assessment Method: Pain and Patients’ Discomfort

In order to assess the pain and patients’ discomfort before and at the end of treatments in all groups, a Visual Analogue Scale (VAS) for the severity of pain sensation was used. 0 represented no pain at all and 100 represented the greatest pain. Each participant was asked to assess his pain from 0 to 100 before the treatment and at 6 weeks of follow-up.

In order to assess the clinical aspect of the painful and erosive OLP at different times of follow-up, the REU score, established by Piboonniyom S et al., was used (Table 2) [13]. The follow-up sessions started after 6 weeks of treatments for both groups.

### 2.7. Assessment of the Recurrence

The recurrence rate of erosive areas was evaluated by comparing the patient’s clinical conditions at the end of the treatment with their clinical conditions before treatment (baseline). No recurrence was considered when the OLP lesion did not present with any new atrophic/erosive lesions. Recurrence was considered when the patient presented with a new atrophic/erosive lesion in the treated site during the follow-up periods. Recurrence was assessed at 6 weeks after the end of each treatment, and again at 3 months, 6 months and 12 months, for all groups.

### 2.8. Statistical Analysis

For the statistical analysis, Prism 5 software (GraphPad Software, Inc., San Diego, CA, USA) was utilized. The confidence level was 95% with a *p*-value < 0.05 considered as statistically significant for the analysis. Descriptive statistics, including the means and standard deviations, were also calculated. Repeated measures and non-parametric ANOVA with a Kruskal–Wallis test coupled to Dunn’s multiple comparison test (post hoc test) were used.

## 3. Results

### 3.1. Pain Assessment

Both corticosteroid and PBM groups showed a significant reduction of VAS scores from 80.65 ± 4.1 and 83.54 ± 3.7 to 0 pain, respectively. Therefore, the treatment can be considered as successful regarding the management of pain. Both treatments improved the quality of patient’s life through the reduction of pain and discomfort (Table 3).

### 3.2. REU Score

After 6 weeks of treatment, there was a significant reduction in the overall REU score for the corticosteroids group and the PBM group from an overall score of 4 to 1 in both groups. Therefore, with six weeks of follow-up, both PBM and corticosteroids showed similar results in terms of REU score without significant difference (Table 4).

### 3.3. Recurrence Rate

**Figure 3:**
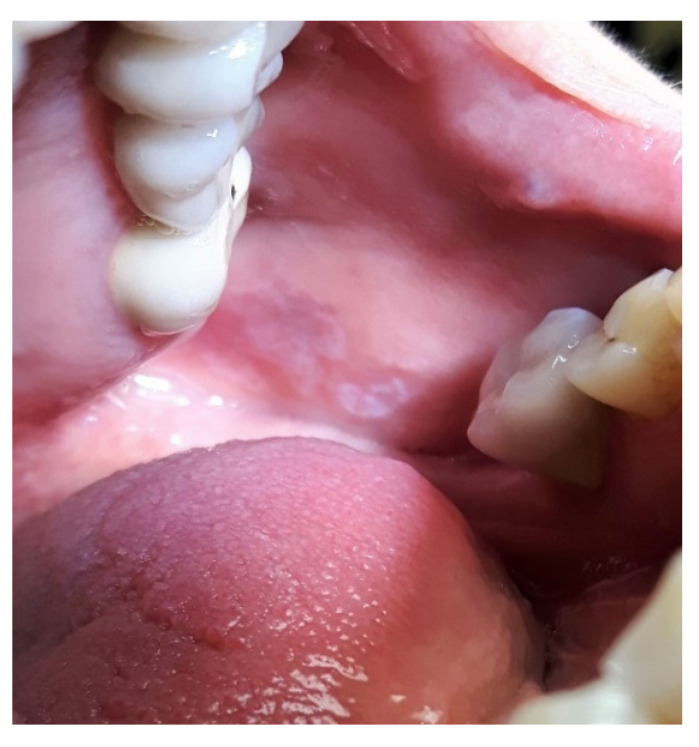
Aspect of the OLP at 6 weeks of PBM treatment. The erosive area and pain have disappeared. A slight reduction in the size of the lesion may be noted.

**Figure 4:**
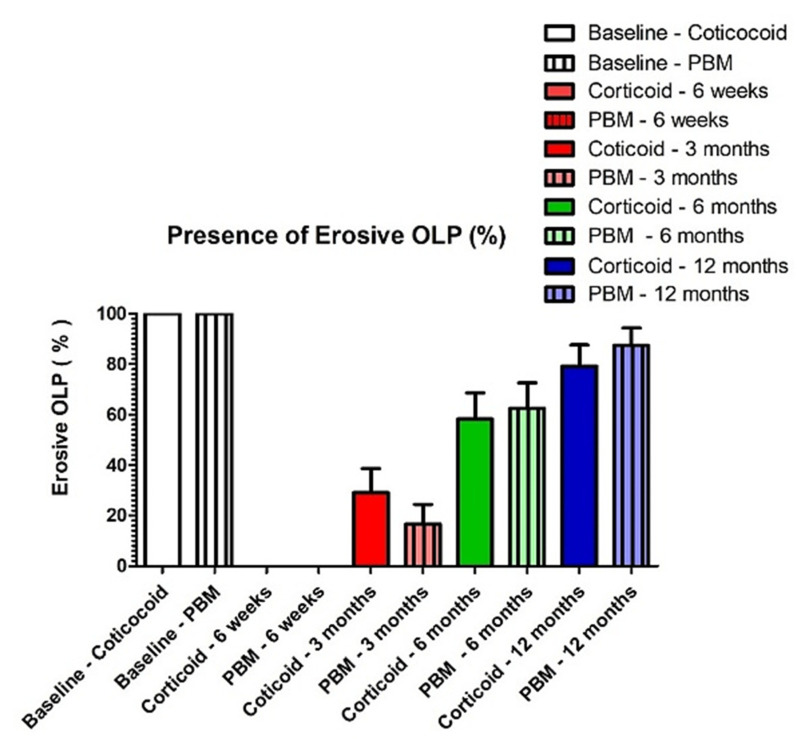
Recurrence rate of erosive and painful OLP at different periods of follow-up.

No significant difference was found between both groups at all timesteps of follow-up in terms of recurrence rate of erosive areas. After 6 weeks, no recurrence was detected (Figure 3) for both groups (0%). The values increased significantly at each time of follow-up (3, 6 and 12 months), and at 12 months, 79% and 87.5% were the recurrence rates for both corticosteroid and PBM groups, respectively, without significant difference between groups [Table 5, Figure 4].

The null hypothesis that PBM therapy will not have a significant impact on OLP was rejected.

## 4. Discussion

In this retrospective study, the conventional application of corticosteroids for erosive and painful OLP offered almost the same stability (without significant difference) when compared to the PBM therapy. Clinically, both PBM and corticosteroids showed a significant, similar result in the management of pain, since all included patients reported no pain after both treatments after six weeks of follow-up. Corticosteroids showed superior stability (less recurrence of erosion) after a year of follow-up; however, this superior result obtained was not significant when compared to the values obtained with PBM.

After interpreting the results, it can be observed that corticosteroids and PBM presented the same results in this study. However, since corticosteroids presents side effects such as stinging, burning, irritation, dryness, or redness [14,15,16], it can be underlined that PBM might be considered as a very promising approach for the treatment of OLP.

OLP is an autoimmune chronic inflammatory disease [5,8] and since PBM was shown to be an effective method for the attenuation of inflammation, the idea of applying PBM was rational to be suggested as an alternative approach for the management of OLP [4,17]. Today, it is well-established that PBM reduces the inflammatory process, accelerates wound healing and tissue regeneration, prevents fibrosis, reduces pain and improves function [4,10,17,18]. These photobiological reactions have been shown to occur in various tissues and were proven by different studies on different pathological conditions to be reliable and predictable if the correct parameters and protocol are applied [4,18,19]. Although there has been significant improvement in understanding PBM’s underlying mechanism of action, the exact mechanism is not fully understood [19]. What is well-known is that PBM acts predominantly on cytochrome c oxidase (CcO) in the mitochondrial respiratory chain by facilitating electron transport, resulting in an increased transmembrane proton gradient that drives adenosine triphosphate (ATP) production [19]. This increase in ATP can enhance bioavailability to power functions of cellular metabolism, since ATP is the energy of living cells. In addition, PBM can cause in stressed cells a short, transient burst of reactive oxygen species (ROS) that is followed by an adaptive reduction in oxidative stress [20]. This modulation of ROS production has been shown to mimic the activity of molecular agents that attenuate tissue damage, such as amifostine, N-acetyl cysteine, and superoxide dismutase [20]. It was also demonstrated that PBM acts on the inflammatory process by causing reduction of inflammation initiators, stimulating the fibroblasts, facilitating the deposition of collagen fibers and rebuilding the extracellular matrix as the wound site occurs [19,20,21,22].

Similar studies were conducted using PBM therapy with the aim of treating OLP [21,22]; however, there is no consent on the exact PBM dosimetry or treatment protocol for the management of erosive OLP. In this context, a randomized double-blind study by Rodrigues et al. [23], showed that the use of PBM can be as effective as corticoid therapy in treating oral lichen planus with no adverse side effects noted. In their study, a 660 nm diode laser was used in a continuous mode with a spot size of 0.283 mm^2^, an output power of 100 mW with a 5 s of exposure time per point and 0.5 J of total energy per point twice a week for 4 weeks and for a total of eight sessions [23]. A systematic review and meta-analysis by Wang et al. [12] argues that although PBM is proving to be a reliable alternative to topical corticosteroids, additional long-term randomized clinical trials and well-designed RCTs with long-term periods are still recommended to consolidate the effectiveness of PBM [12]. On the other hand, a study using an optical coherence tomography was made in patients with atrophic-erosive oral lichen planus treated by PBM (study group) and 0.05% clobetasol propionate (control group) for 8 weeks [24]. This study by Gambino et al. [24] concluded that with PBM and clobetasol propionate an increase in the width of stratified epithelium and decrease in lamina propria can be observed. It was also concluded by Gambino et al. [24] using optical coherence tomography that clobetasol provides more significant short-term structural changes, whereas PBM guarantees long-term alteration [24].

Besides PBM, photodynamic therapy is also showing promising results for managing oral complications including OLP [25]. Photodynamic therapy (PDT) is based on the use of a photosensitizer (dye, photoactive agent) that is activated by a specific wavelength of light [26]. The photosensitizer interacts exclusively with diseased cells. After photoactivation, the photosensitizer releases free radical derivatives toxic to the targeted tissue, resulting in targeted and selective destruction and necrosis of this pathological tissue [26]. In this context, a meta-analysis by Yuqing He et al. [25] showed that PDT is effective for the management of OLP and can be a second option in cases of resistance to corticosteroids. According to this meta-analysis, after PDT therapy, the size of OLP lesions decreased depending on their baseline size, and pain also decreased significantly after PDT. In addition, they showed that the photosensitizer 5-aminolevulinic acid (5-ALA) was more effective than methylene blue (a frequently used photosensitizer) [25].

Our study confirmed that the gold standard treatment for painful and erosive OLP remains the topical application of corticosteroid. In addition, our results showed that PBM offered complete disappearance of pain and erosive/ulcerative lesions of OLP after six weeks of treatment. It was, remarkably, observed that PBM therapy showed significantly similar results in terms of pain management and recurrence rates when compared to the standard of care. This may be very promising, as PBM has no reported side effects, such as the well-documented long-term and short-term side effects of topical corticosteroid application. However, further future studies using our treatment procedure with a higher number of patients are needed to confirm the effectiveness of PBM for the treatment of erosive and painful OLP.

## 5. Conclusions

Within a follow-up period of one year, this retrospective study showed that PBM is an effective therapy. Moreover, the success and recurrence rates were similar, with no significant difference, to the conventional topical application of corticosteroid in the management of erosive/ulcerative OLP. Hence, PBM can be considered as a promising approach with no reported side effects for the management of OLP.

## Figures and Tables

**Table 1:** Clinical features of the treated patients.

Total Participants	Gender		Mean Age Range (Years)	Average Size of OLP (cm^2^)
96	Female	Male	48 (min 42; max 68)	2.2 (min 1.5; max 3.4)
	64	32		

Age in years; average size in centimeter square (cm^2^); min= minimum; max= maximum.

**Table 2:** Scores of REU signs of oral lichen planus [13].

Clinical Signs	Score of Signs
Reticulate/plaque-type (R)	0 = none
	1 = white streaks or patches
Congestive/atrophic (E)	0 = none
	1 = lesions < 100 mm^2^
	2 = lesions 100 mm^2^ to 300 mm^2^
	3 = lesions > 300 mm^2^
Ulceration (U)	0 = none
	1 = lesions < 100 mm^2^
	2 = 100 mm^2^ to 200 mm^2^
	3 = lesions > 300 mm^2^

**Table 3:** VAS values before treatment and at 6 weeks of follow-up. 0 represented no pain at all and 10 represented the greatest pain. Similar letters indicate non-significant differences. Different letters indicate significant differences.

	Before Treatment	At 6 Weeks of Follow-up
Corticosteroid group	80.65 ± 4.1 ^a^	0 ^b^
PBM group	83.54 ± 3.7 ^a^	0 ^b^

**Table 4:** Total values of the REU score for oral lichen planus before and at the end of treatment for each group. Similar letters indicate non-significant differences. Different letters indicate significant differences.

	REU Score	
	Before Treatment	After 6 Weeks of Treatment
Corticosteroid group	R = 1 E = 2 U = 1	R = 1 E = 0 U = 0
	Overall score 4 ^a^	Overall score 1 ^b^
PBM group	R = 1 E = 2 U = 1	R = 1 E = 0 U = 0
	Overall score 4 ^a^	Overall score 1 ^b^

**Table 5:** Recurrence rate of erosive & painful OLP at different periods of follow-up. Similar letters indicates non-significant differences. Different letters indicate significant differences.

	6 Weeks	3 Months	6 Months	12 Months
Corticosteroid group (*n* = 48)	0% ^a^	29% ^b^ (14 of 48)	58% ^c^ (28 of 48)	79 % ^d^ (38 of 48)
Photobiomodulation group (*n* = 48)	0% ^a^	21 % ^b^ (10 of 48)	62 % ^c^ (30 of 48)	87.5 % ^d^ (42 of 48)

## Data Availability

The data presented in this study are available from the corresponding author upon request.

## Abbreviations

PBM = photobiomodulation; OLP = Oral lichen planus; CW = continuous wave; NAALT = The North American Association of Laser Therapy; WALT = World association for laser therapy; LP = Lichen planus; WHO = world health organization; VAS = visual analogue scale; PDT = photodynamic therapy; ATP = adenosine triphosphate; ROS = reactive oxygen species.
